# Porous biomaterial scaffolds for skeletal muscle tissue engineering

**DOI:** 10.3389/fbioe.2023.1245897

**Published:** 2023-10-03

**Authors:** Natalie G. Kozan, Mrunmayi Joshi, Sydnee T. Sicherer, Jonathan M. Grasman

**Affiliations:** Department of Biomedical Engineering, New Jersey Institute of Technology, Newark, NJ, United States

**Keywords:** volumetric muscle loss, biomaterial, tissue engineering, porosity, scaffold, skeletal muscle, skeletal muscle tissue engineering

## Abstract

Volumetric muscle loss is a traumatic injury which overwhelms the innate repair mechanisms of skeletal muscle and results in significant loss of muscle functionality. Tissue engineering seeks to regenerate these injuries through implantation of biomaterial scaffolds to encourage endogenous tissue formation and to restore mechanical function. Many types of scaffolds are currently being researched for this purpose. Scaffolds are typically made from either natural, synthetic, or conductive polymers, or any combination therein. A major criterion for the use of scaffolds for skeletal muscle is their porosity, which is essential for myoblast infiltration and myofiber ingrowth. In this review, we summarize the various methods of fabricating porous biomaterial scaffolds for skeletal muscle regeneration, as well as the various types of materials used to make these scaffolds. We provide guidelines for the fabrication of scaffolds based on functional requirements of skeletal muscle tissue, and discuss the general state of the field for skeletal muscle tissue engineering.

## 1 Introduction

Volumetric muscle loss (VML) is a condition whereby a loss of skeletal muscle results in impairment of both the regenerative capacity and overall functionality of the muscle. Even a small (10%–20%) loss of muscle weight can result in an overall 30%–90% loss of strength (Corona et al., 2015). Skeletal muscle possesses the ability to regenerate small-scale injuries through the recruitment and differentiation of satellite cells (SCs), the local progenitor cell of skeletal muscle tissue. However, once too much muscle mass has been destroyed, the tissue loses its regenerative capabilities (Grasman et al., 2015b). It is estimated that 65.8 million Americans sustain musculoskeletal injuries each year, including VML and other soft tissue injuries, and the treatment cost of these injuries surpasses 176 billion U.S. dollars (Carnes and Pins, 2020b). This loss of muscle can result from traumatic events such as from surgery, cancer resection, car crashes, or battlefield injuries (Grogan et al., 2011). VML is common amongst battlefield injuries; it has been found that 54% of soldiers who have sustained an injury from the battlefield suffer from musculoskeletal injuries and from that number, 53% involve damage to soft tissue. In the population of military personnel who have been discharged because of a muscle-related condition, over 90% experienced an injury resulting in VML (Corona et al., 2015).

The standard of care for VML repair is an autologous tissue transfer. In this treatment, an undamaged muscle flap is surgically removed and grafted into the VML site. This treatment method has several limitations. First, the grafted muscle flap cannot fully restore lost functionality of the damaged muscle. Autologous tissue transfer may also result in tissue necrosis or infection, which occurs in about one out of ten of these procedures (Mulbauer and Matthew, 2019). Additionally, this treatment requires complicated surgery and thus demands a high level of surgical skill, resulting in limited access to this treatment based on location and surgeon availability (Carnes and Pins, 2020b). Another treatment that is commonly administered along with autologous tissue transfers is physical therapy (rehabilitation). Physical therapy strengthens the muscle groups that remain after injury and has been shown to enhance angiogenesis, regulate the immune response, and the release of myogenic growth factors (Liu et al., 2018). Although physical rehabilitation can significantly improve the functionality of injured muscle, it cannot induce large amounts of muscle regeneration. Thus, where large volumes of muscle are lost, the patient is limited in the types of exercises they can actually participate in for physical therapy (Liu et al., 2018). Additionally, functional deficits caused by VML can ultimately lead to late-stage amputation of the injured limb (Sorensen et al., 2022). Therefore, there is a clear need to develop tissue engineering techniques for skeletal muscle regeneration which can restore injured muscle volume and functionality. To enable broader utilization of tissue engineered treatments, there should be a material or approach which can be more easily administered without the need for advanced surgical skill.

In this review, we discuss various strategies to develop biomaterial scaffolds for use in treatment of VML and other skeletal muscle pathologies by mimicking the skeletal muscle tissue niche. The goal of this review is to highlight several important design criteria which need to be considered when fabricating a scaffold for this purpose, the most important being scaffold porosity and alignment. We will also discuss methods of scaffold fabrication as well as summarize potential materials used in the development of these scaffolds. Finally, we discuss the importance of scaffold optimization and provide future strategies and requirements to successfully fabricate scaffolds for use in skeletal muscle tissue regeneration.

## 2 Skeletal muscle anatomy

**FIGURE 1:**
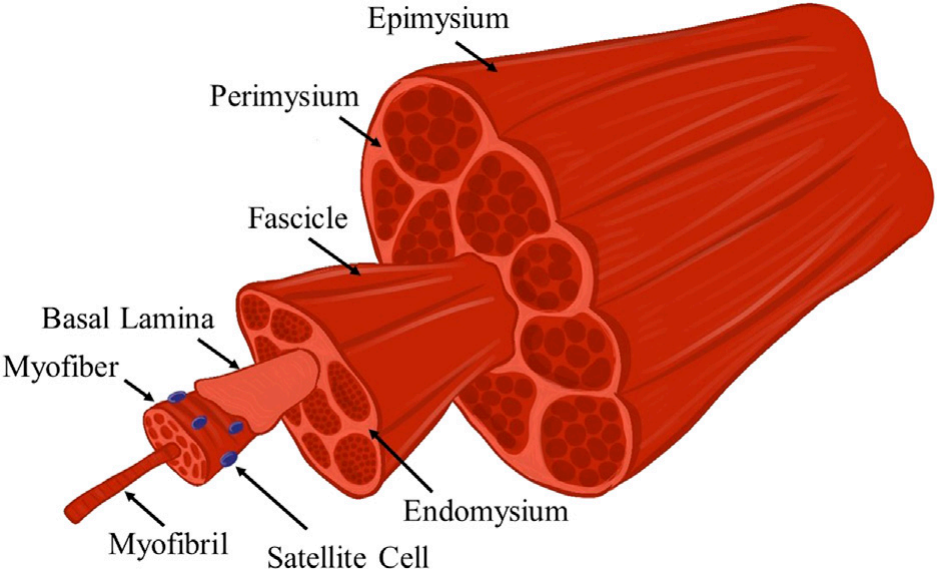
Schematic illustration of the anatomy of skeletal muscle tissue. This hierarchal arrangement of myofibers of increasing size-scales is responsible for the efficient transmission of force. Several notable anatomic features that are relevant to this review are highlighted.

Skeletal muscle is a highly aligned tissue, the anatomy of which is highlighted in Figure 1. This tissue is made up of long fibers called myofibers, which upon stimulation from the neuromuscular junction, synchronously contract to generate force, which is used for a variety of functions including locomotion and ambulation of peripheral limbs. Each myofiber within a muscle is covered by the basal lamina, which is made up of several proteins such as type IV collagen, laminin, and fibronectin. Glycosaminoglycans (GAGs) such as heparan sulfate are also contained within the basal lamina. SCs are found in the space between the myofiber and the basal lamina, and are stimulated to regenerate damaged tissues based on growth factors and mechanical signals transmitted by the basal lamina and GAGs such as heparan sulfate (Velleman, 1999; Grasman et al., 2015b). Each fiber is comprised of multiple myofibrils and is surrounded by the endomysium. The endomysium is a highly ordered, load-bearing network that surrounds muscle fibers and aids in force transmission (Gillies and Lieber, 2011; Sharafi and Blemker, 2011). Bundles of muscle fibers are known as fasciculi, and are covered by the perimysium, which is comprised of organized collagen (Gillies and Lieber, 2011). The epimysium surrounds the whole muscle and is made up of large, crimped collagen bundles which aid in force propagation across the myotendinous junction (Gillies and Lieber, 2011; Dave et al., 2022).

When considering how to best develop biomaterial scaffolds for use in skeletal muscle tissue engineering, one of the most important attributes of skeletal muscle, in addition to its ability to generate and transmit forces, is its alignment. A biomaterial scaffold must mimic the aligned nature of skeletal muscle to facilitate the growth of fibers and restore the damaged muscle. The scaffold should also support the infiltration of many cells, direct SC fusion and differentiation to form new myotubes, and facilitate nascent myotube maturation into myofibers and overall incorporation into the healthy host tissue. Another important characteristic of skeletal muscle is its ability to withstand dynamic loading, which is in part modulated by its mechanical properties. Therefore, successful scaffolds for skeletal muscle regeneration must facilitate cellular ingrowth and alignment while being mechanically stable within a mechanically actuating tissue.

### 2.1 Skeletal muscle regeneration

Skeletal muscle maintains an innate ability to regenerate after injury. Regeneration requires several different processes, such as infiltration of cells, vascularization, innervation, and differentiation of muscle progenitor cells into myofibers and is broken down into three stages: the destruction/inflammation phase, the repair phase, and the remodeling phase. This process of regeneration, particularly the repair phase, can occur as long as the basal lamina remains intact. The basal lamina is necessary as it acts as a scaffold for the formation of myofibers and it is able to minimize fibrosis (Grounds, 1991).

In the destruction/inflammatory phase, damaged muscle fibers and necrotic cells get degraded (Grasman et al., 2015b). Degradation of the injured myofiber occurs once a specific region of the myofiber becomes necrotized. The propagation of necrosis along the myofibers is stopped by a contraction band, which is formed by cytoskeletal contraction at the site of damage within the fiber (Exeter and Connell, 2010). The gap between the two ends of the necrotized myofiber then gets filled with a hematoma, which ultimately induces an inflammatory reaction (Järvinen et al., 2013), facilitating the infiltration of neutrophils and macrophages which degrade the damaged fiber (Grasman et al., 2015b; Ma et al., 2018). These cells also release inflammatory factors such as TNF-α, which inhibit myogenic cell differentiation during this phase of acute inflammation and proteolytic activity (Ma et al., 2018).

In the repair phase, SCs are recruited to the injury site. Upon activation, SCs will re-enter the cell cycle and a population of them will commit to differentiation, becoming myoblasts. These undifferentiated myoblasts proliferate further to increase cell density, and will ultimately fuse with one another to form myofibers. Within approximately 5 days, the necrotized myofiber will be replaced by newly formed myofibers. In this phase, capillaries also begin to grow into the injury site to aid in vascularization (Järvinen et al., 2013), and growth factors such as insulin-like growth factor 1 (IGF-1) are upregulated to stimulate myogenesis and myofiber growth (Ma et al., 2018).

The remodeling phase is the phase in which regenerating myofibers mature, forming new contractile units, and integrate with the surrounding healthy tissue (Järvinen et al., 2013). Innervation is also essential for the maturation of myofibers; the formation of neuromuscular junctions (NMJs) is necessary for muscle functionality. Newly formed NMJs are typically observed within 2–3 weeks after muscle damage (Laumonier and Menetrey, 2016). Another characteristic of the remodeling phase is that in this phase, fibroblasts infiltrate the wound site and repair the damaged connective tissue in the area. In larger injuries, fibroblasts may remodel the connective tissue into scar tissue, thus limiting the functional capabilities of this tissue.

In VML, there is a lack of myoblast infiltration due to lack of regenerative cues from the basal lamina, resulting from the traumatic loss of tissue (Grasman et al., 2015b). These injuries have an adjusted destruction process, as the tissue at the margins of the injury are degraded rather than the entire injury site (Downing et al., 2021). Instead of regeneration, persistent inflammation occurs at the site of the VML injury. This inflammation inhibits satellite cell migration into the defect site, reducing the amount of *de novo* muscle tissue formation, and rather induces fibro-adipogenic progenitors to migrate, proliferate, and differentiate to form fibroblasts. These fibroblasts deposit a fibrotic matrix at the injury site, resulting in lack of functionality of the muscle (Larouche et al., 2018). Therefore, there is a need for a treatment which can bring back the lost regenerative cues, stimulate myoblast infiltration, and rehabilitate the injured tissue.

## 3 Design requirements for biomaterial scaffolds

**FIGURE 2:**
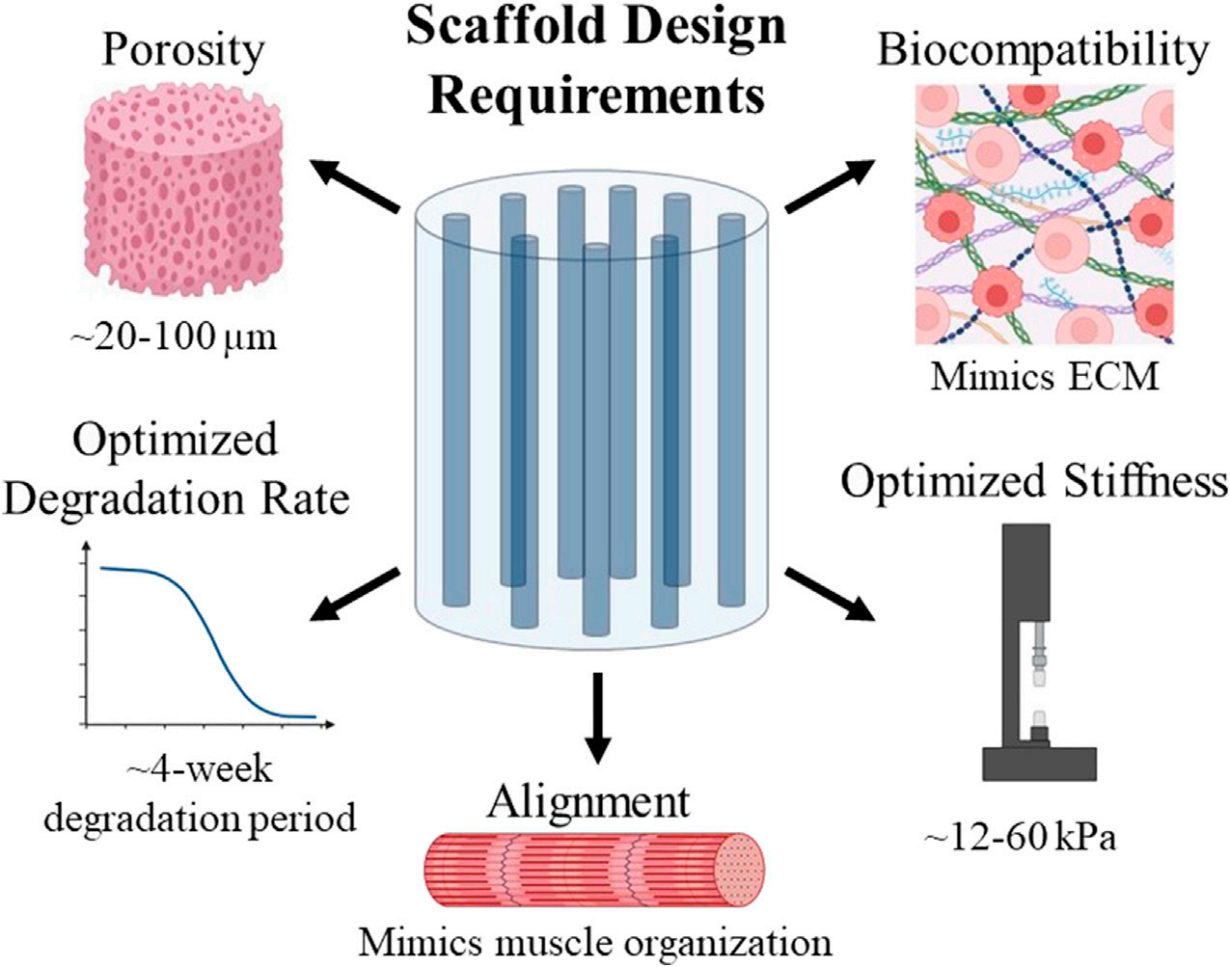
Summary of design requirements to be considered when creating scaffolds for use in skeletal muscle tissue engineering.

Many types of biomaterials are currently being tested for use in skeletal muscle regeneration. The goal for biomaterial design is to create a material that can be implanted into the injury site and will regenerate the tissue. Biomaterial scaffolds can be comprised of natural biomaterials, such as collagen, fibrin, alginate, or decellularized ECM, or synthetic biomaterials (Eugenis et al., 2021), and several important design criteria for skeletal muscle tissue engineering are summarized in Figure 2. The scaffold must also facilitate alignment and must allow for cell infiltration and nutrient transfer. To achieve this, scaffold porosity can be modulated to allow for cell migration into the scaffold. Alignment in a scaffold can refer to the lining up of material comprising the scaffold—*e.g.*, the alignment of collagen fibers. It can also refer to the pores within the scaffold being lined up in a directional manner, creating a channel-like structure. Channels within a scaffold provide an environment conducive to the formation of aligned myofibers (Jana et al., 2016). Scaffold alignment is critical for linear orientation of myoblasts, for their subsequent fusion into myofibers, and ultimately for their ability to efficiently contract to generate force (Cheng et al., 2020). Functional outcomes depend on the ability of the regenerated muscle to produce mechanical forces, which can be dependent on the angle of pennation within the specific muscle body (Westman et al., 2019; Sicherer et al., 2020). Therefore, we posit that controlling the alignment of scaffolds to mimic this organization will properly produce these forces and maximize regeneration. The pores within a scaffold should have certain characteristics such as a size which supports the influx of cells and interconnectivity which allows for nutrient transfer.

Another characteristic of skeletal muscle to consider when designing a scaffold is its mechanical properties, namely stiffness values. The stiffness of native skeletal muscle tissue ranges from 12–16 kPa (Jana et al., 2016; Chaturvedi et al., 2017). Scaffolds for skeletal muscle tissue engineering must have a similar stiffness; if a scaffold is too stiff, it will not be able to transfer load to the cells within it since it cannot deform (Breuls et al., 2008). Matching the scaffold stiffness to the tissue stiffness is necessary to transmit forces to these cells in order to influence their fate (Jana et al., 2016). If a scaffold is too soft, myotube formation can be inhibited (Jana et al., 2016). Therefore, the stiffness of a scaffold should mimic that of native skeletal muscle tissue to maximize biocompatibility and myogenic differentiation to form new myofibers which can integrate into healthy tissue.

Degradation rate must also be considered when designing a biomaterial scaffold. After implantation, biomaterial scaffolds should eventually degrade to be replaced by native tissues. The rate of degradation of a scaffold should ideally be equal to the pace of ECM generation to best facilitate muscle regrowth, as ECM forms gradually (Kheradmandi et al., 2016). Degradation of a scaffold is also important because the degradation products of a biomaterial scaffold are often bioactive, although the functions of these degradation products can vary between having antimicrobial effects, chemotactic effects for a variety of cell types, or they can even modulate the host immune response (Badylak et al., 2016). Because skeletal muscle injuries often take within 2–6 weeks to heal (Quint et al., 2022), the degradation of a scaffold used for skeletal muscle tissue regeneration should ideally be modulated to match this timeline.

### 3.1 Porosity

Porous scaffolds are beneficial in the field of tissue engineering because they facilitate nutrient/gas exchange, and increase the surface area with which cells attach, proliferate, and differentiate. The porosity, and interconnectivity, of pores within the scaffold allows cells to infiltrate through these spaces to facilitate tissue regrowth throughout the scaffold. In their native configuration within the body, the space between cells is filled with the ECM. Glycosaminoglycans, a major component of the ECM, form porous hydrated gels which fill up most of the space around cells (Alberts et al., 2002). While porosity within a tissue in the body can be defined as space either filled with these hydrated molecules or within a gel formed by the hydrated molecule, we define porosity, for the purpose of this review, as the negative space between the insoluble content of the scaffold. It is critical to note the importance of hydration for natural scaffolds, as this will impact the presentation of the porous structure, while synthetic scaffolds will not produce as much of a difference in the porous structure in their hydrated state. In addition to fabricating tissue, porosity within a scaffold also creates space for secondary structures, such as blood vessels, and it leads to uniform degradation of the scaffold (Qazi et al., 2015). Modulation of porosity and interconnectivity of pores within a scaffold can impact mechanical properties such as compressive modulus (Haas et al., 2019). Control of the pore size in sponges can facilitate varying amounts of cell viability, cell differentiation, and nutrient diffusion (Carnes and Pins, 2020b). Pore size has also been shown to influence macrophage polarization; macrophages within a scaffold with 34 µm pores had a 63% increase in M1 polarization and an 85% decrease in M2 polarization as compared to macrophages cultured outside of the porous scaffold (Sussman et al., 2014). The porosity within a scaffold can also be categorized as homogeneous or heterogeneous. In heterogeneous scaffolds, the level of porosity, pore size, shape, and location are manipulated to optimize these characteristics for a specific application. Heterogeneous pore structures may be beneficial to develop a controlled distribution of cells and growth factors throughout the scaffold, as varying the shape of the pores has been shown to affect cell growth (Yoo, 2012; Khoda and Koc, 2013). Heterogeneous pores, however, may negatively affect the mechanical strength of the scaffold (Civantos et al., 2020). Polymeric scaffolds with homogeneous, uniform pore distributions have improved mechanical properties (Kim et al., 2005). Therefore, further research should seek to determine the effects of homogeneous *versus* heterogeneous pore structure on myoblast behavior. There is a need for porous substrates for skeletal muscle tissue engineering because myoblasts within the scaffold must be able to aggregate in close enough proximity so that they can fuse to form myotubes. The newly formed tissue needs to have a synchronized response with the host to facilitate efficient transmission of force, which is made possible by aligned aggregation of cells and myofibers (Kheradmandi et al., 2016).

Porous biomaterial scaffolds have shown several indicators which suggest that they promote long-term regeneration of skeletal muscle tissue. In one study, the efficacy of non-porous scaffolds was compared to that of porous scaffolds fabricated from polycaprolactone (PCL) and decellularized muscle ECM in the context of skeletal muscle regeneration. These scaffolds were tested *in vitro* by seeding the scaffolds with induced myogenic progenitor cells. The porous, fibrous scaffolds were able to support the most myogenic cell fusion, as quantified by myosin heavy chain (MHC) expression and fusion index analysis, out of all the experimental groups. After implantation, not only did these scaffolds facilitate skeletal muscle tissue regeneration, but they also supported the formation of CD31-positive capillaries, indicating revascularization. Non-porous scaffolds showed lower myogenic gene expression and lower fusion index than porous scaffolds, and thus were not implanted *in vivo*, clearly demonstrating the utility of porosity for muscle regeneration (Jin et al., 2021b).

### 3.2 Methods of fabrication of porous scaffolds

**FIGURE 3:**
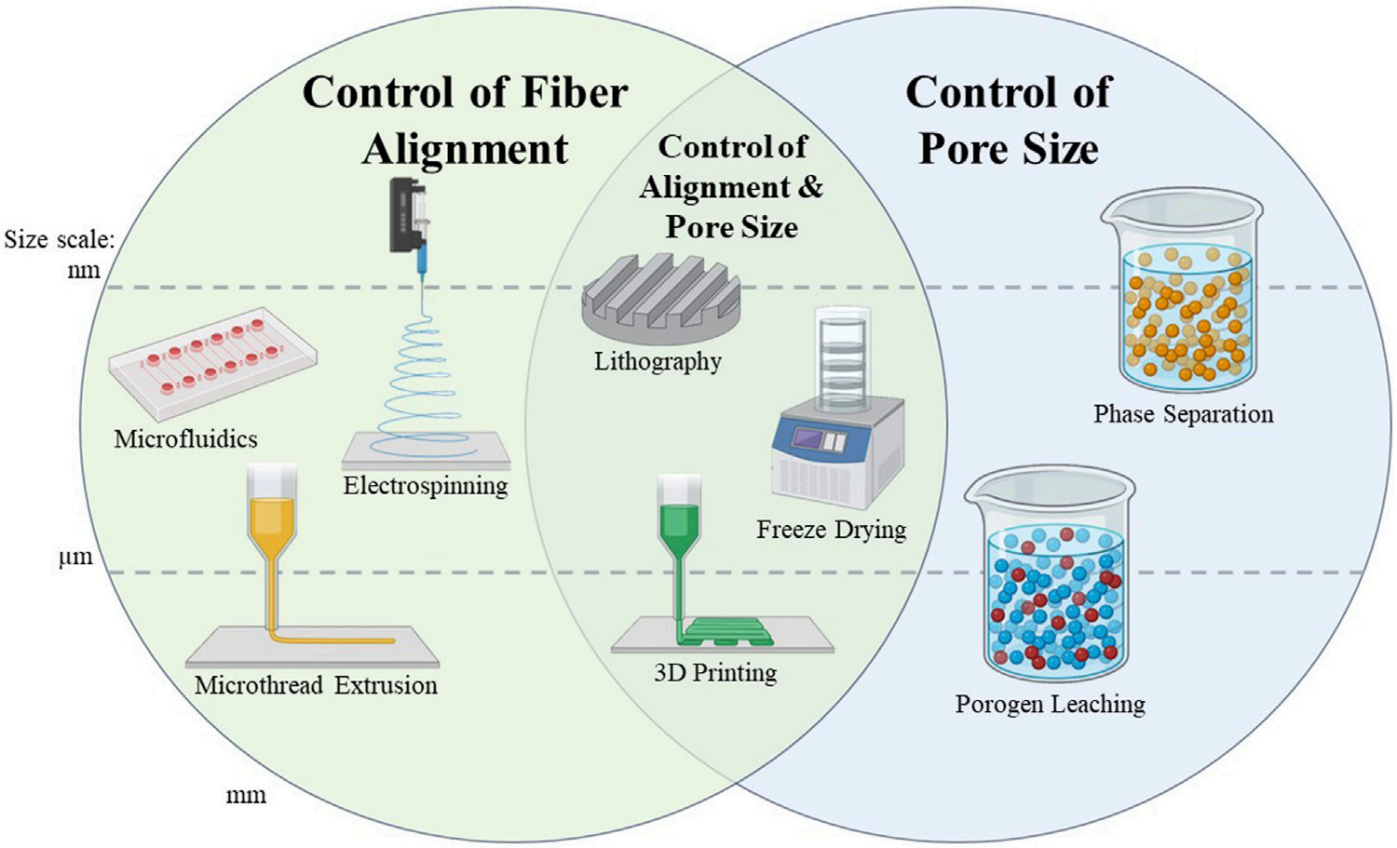
Summary of scaffold fabrication methods capable of controlling scaffold alignment and/or pore size. Methods are separated into the size scale at which the fibers or the pores of the scaffold can be created. These size scales are separated into the nanometer, micrometer, and millimeter scale. Methods which cross the dotted lines can be used to create scaffolds with features at multiple size scales, which are indicated by placement of the graphic.

There are various methods which can produce scaffolds with porous architectures, such as electrospinning, porogen leaching, and freeze-drying (Figure 3). Many materials have been electrospun into scaffolds for skeletal muscle regeneration; examples of which are poly (lactic-*co*-glycolic acid) (PLGA), PCL, collagen, elastin, gelatin, hyaluronic acid, and silk fibroin. These materials have displayed potential for use in skeletal muscle tissue engineering (Klumpp et al., 2010; Narayanan et al., 2016; Beldjilali-Labro et al., 2018; Politi et al., 2020). Electrospinning is an effective method for biomaterial fabrication because it creates ECM-like fibers. There are numerous processing parameters during the electrospinning process that can be tuned to create scaffolds with various characteristics. For example, electrospinning can produce random or aligned scaffolds depending on the collector geometry and rotational speed, and fiber thickness can be varied based on the concentration of the polymer being used or the distance of the nozzle to the collector plate (Beldjilali-Labro et al., 2018). The type of nozzle, collector surface, flow rate, and applied voltage can also vary the resulting material properties (Kulkarni et al., 2010; Vass et al., 2020). Pore morphology can be controlled by changing the biomaterial/solvent ratio, using a sacrificial template, or conducting other post-processing methods (Wang et al., 2023). Internal porosity can be created within the electrospun fibers by using various phase separation techniques, in which solvents can be separated out to leave pores behind in the material (Huang and Thomas, 2020). Although the electrospinning process is useful for creating porous scaffolds and can be used to fine-tune scaffold architecture, it remains challenging to fabricate nanofibrous scaffolds with precise dimensions and morphology (Pant et al., 2023).

3D printing techniques can also be used to fabricate porous scaffolds by designing specific architectures within the structure at the micro and/or macro levels (An et al., 2015; Chia and Wu, 2015; Jammalamadaka and Tappa, 2018; Yan et al., 2018). Solid freeform fabrication (SFF) includes processes in which materials are created from computer-aided design files without using molds or other forming tools (Trunec and Maca, 2014). Such strategies are attractive for tissue engineering because this technology can be used to make 3D structures that are customized to the needs of each patient, and gives the user direct control over scaffold architecture (Chia and Wu, 2015). One of the major limitations of bioprinting is the lack of consensus regarding printing parameters. Since the composition of bioinks, printing parameters, and biofunctionalization methods vary greatly, it is difficult to determine the gold standard for a specific tissue type (Sigaux et al., 2019). The degradation rates of bioinks also differ from those of native tissue—bioinks tend to degrade much slower in part because of the stabilization processes required to endure fabrication (Parak et al., 2019). Improvements in aligning bioink degradation rate to that of the formation rate of tissue at the repair site are being explored (Jeon et al., 2019; Singh et al., 2019; Barceló et al., 2022). 3D bioprinting has been explored for use in skeletal muscle regeneration, showing promise with the use of materials such as alginate, gelatin, fibrin, and collagen, and cell types such as C2C12s, fibroblasts, and human umbilical vein endothelial cells (HUVECs) (Zhuang et al., 2020).

Another common method to create a porous scaffold is through the incorporation of a porogen, such as salt or sugar molecules, into solution with the biomaterial. Incorporation of the porogen occurs immediately prior to the polymerization of the scaffold, such that the scaffold forms around the porogens. Once the scaffold is fully formed, porogens are removed by incubation in an appropriate solvent, resulting in the formation of pores interspersed throughout the scaffold (Chen et al., 2002; Lee et al., 2005; Huri et al., 2014). The size of the porogen directly determines the size of the pores in the scaffold, and the concentration of the porogen within the scaffold determines its porosity. Porogen leaching is an effective method for creating porous scaffolds, since it provides direct control over both the size and distribution of the pores (Chen et al., 2002). This method is thus highly tunable to create scaffolds with specific pore sizes and porosities. Porogen leaching does present some limitations, however. The use of large amounts of salt porogens in a material can reduce the mechanical strength of the material once the porogen is removed, as indicated by a decrease in compressive modulus. Additionally, inefficient removal of the porogen can result in local regions of the scaffold with high osmolarity, leading to local toxicity (Kim et al., 2009b).

Freeze-drying is a process that uses ice as a porogen. Polymers are frozen and then lyophilized to remove ice crystals, resulting in an interconnected, porous architecture. In this process, a protein scaffold is first formed using water as a solvent, frozen, and lyophilized to remove the ice and leave a porous structure within the scaffold. When the solution is frozen, ice crystals form, causing the biomaterial to orient around the ice crystals. Sublimation of the ice crystals leaves behind a network of interconnected pores which can act as a scaffold for use in tissue engineering applications (Freyman et al., 2001). Freeze drying can be limited by potential heterogeneity of pore architecture and by small pore sizes, however these parameters can be tuned by adjusting the rate of freezing as well as bulk polymer concentration (Deville et al., 2006; Perez-Puyana et al., 2020). Freeze drying has several benefits as compared to other scaffold fabrication methods. Freeze drying uses water as a solvent, which is preferred over the use of harsh chemical solvents, as are often used in fabrication methods such as electrospinning. Additionally, freeze drying can be combined with other methods such as gel casting and salt leaching to improve scaffold properties (Feroz and Dias, 2021). For the remainder of this review, we will focus on scaffold formulations utilizing this principle.

### 3.3 Control of pore size

Another important aspect of porous biomaterial scaffolds is the size of the pores (Figure 3). Larger pores (in the range of a few hundred microns) are beneficial for the infiltration of cells (Phipps et al., 2012), while smaller pores (from nanometers up to several microns) have a high surface to volume ratio, which improves the adsorption of proteins, such as albumin, onto the scaffold. The interaction of adsorbed proteins with cells may facilitate improved regeneration of the tissue (Li et al., 2012; Perez and Mestres, 2016). The ability of small pores (34 µm vs. 160 µm) to control macrophage polarization can also aid tissue regeneration (Sussman et al., 2014). Pores on the 500 µm size scale have been demonstrated to facilitate cell infiltration and differentiation, while 200 µm pores promoted differentiation (Perez and Mestres, 2016). Larger pores (325 vs. 85 µm) tend to promote cell attachment more so than small pores (Murphy et al., 2010). Clearly, pore size can play a major role in directing cell function and behavior.

Controlling the freezing rate of scaffolds while using the freeze-drying method will directly control the size of the resultant pores through a phenomenon known as undercooling. Undercooling is the difference between the temperature of the material during the freezing process and the actual freezing temperature (O’Brien et al., 2005). Larger undercooling facilitates the generation of smaller ice crystals, resulting in smaller pores. Therefore, we can control pore size within a scaffold by varying the temperature at which the material is frozen (O’Brien et al., 2004). In a study by Murphy et al., collagen-GAG scaffolds were produced with mean pore sizes of 96–151 µm. This range was achieved by varying the freezing temperature between −40 and −10°C and using a constant cooling rate to reach the final freezing temperature. Scaffolds with smaller pores (*i.e.,* scaffolds frozen at −40°C) supported significantly more cell attachment than scaffolds with larger pores (*i.e.*, scaffolds frozen at higher temperatures) (Murphy and O'Brien, 2010). A study by Haugh et al. demonstrated that pore size within collagen sponges decreased until the final freezing temperature reached −50°C. Further reductions in the final freezing temperature to −70°C did not create any further decrease in pore size (Haugh et al., 2010). Variation of pore size is important as different types of cells are different sizes and will need different sized pores to mimic their natural extracellular matrix (ECM).

An alternate approach for modifying the size of pores within a biomaterial sponge using the freeze-drying method is changing the concentration of the biomaterial. The higher the biomaterial concentration used, the smaller the resulting pores will be. This has been demonstrated using gelatin (Takemoto et al., 2008; Wu et al., 2010), alginate (Shapiro and Cohen, 1997), chitosan (Ikeda et al., 2014), and collagen (Madaghiele et al., 2008; Yamamoto et al., 2015). Concentration can be used as a control point for pore size because increasing biomaterial concentration will increase the viscosity of the solution. Higher viscosity creates an environment which restricts the growth of ice crystals, creating smaller pore sizes (Wu et al., 2010). Wang et al. demonstrated this with hyaluronic acid (HA), where 0.5% *w/v* scaffolds resulted in pores with an average diameter of 80 μm, which is slightly larger than the diameter of cultured skeletal muscle myoblasts (∼20–30 µm) and improved overall cell attachment. Increasing the amount of HA to 1% *w/v* resulted with pores that were too small for myoblast migration (Wang et al., 2009). A pHEMA-gelatin porous scaffold (2% *w/v* gelatin and a 1:2 ratio of PEGDA:pHEMA) contained pores with an average size of 50–80 μm C2C12 myoblasts adhered to, and proliferated, throughout these scaffolds and formed multinucleated myofibers. These results demonstrate that this pore size range is beneficial for myofiber formation (Singh et al., 2010). The size of the pores within the scaffold must be the correct size to support myofiber growth.

### 3.4 Pore alignment and connectivity

Anisotropy is essential for skeletal muscle regeneration, as skeletal muscle is a highly aligned tissue, and can be controlled through fiber alignment and pore size (Figure 3). Scaffolds made of multilayers of patterned materials act as anisotropic scaffolds which can be used in tissue regeneration. Micro- and nanofibrous scaffolds as well as scaffolds containing aligned pores are common types of scaffolds used in skeletal muscle tissue engineering. Micropatterned substrates with aligned topography can also be used to develop muscle constructs (Jana et al., 2016). Anisotropy can also be induced through electrospinning (Liu et al., 2018; Rezvani Ghomi et al., 2022), lithography (Norman and Desai, 2006), and 3D printing (Zieliński et al., 2023). In the context of porous, sponge-like materials, anisotropy refers to the alignment of the porous network within a scaffold, while an isotropic network would be defined as a uniform distribution of pores with no alignment or inherent connectivity. Channels made of these aligned, interconnected pores may facilitate the formation of aligned myofibers that would integrate with mature host muscle tissue. Myogenic cells seeded onto aligned, nanofibrous poly (hydroxybutyrate) (PHB) scaffolds revealed higher fusion index, myofiber length, and degree of alignment as opposed to cells seeded on non-aligned scaffolds comprised of the same material (Ricotti et al., 2012). A study by Jana et al. looked at 2D anisotropy of fibers within a chitosan-PCL film *versus* a chitosan-PCL nanofibrous substrate. The nanofibrous substrate promoted alignment and elongation of myoblasts, as well as the expression of myogenic differentiation markers after 6 days of culture. These results are in stark contrast with those from the chitosan-PCL films without nanofibrous architecture, which promoted neither terminal differentiation of the myoblasts, nor myoblast alignment (Jana et al., 2014). Together, these results highlight the importance of alignment and demonstrate its importance to aid in the formation and regeneration of skeletal muscle tissue.

### 3.5 Methods of fabrication of aligned porous scaffolds

A facile method to control pore formation within these scaffolds utilizes a controlled or uncontrolled freezing rate. When a sample is fully submerged into a freezing environment, quench freezing occurs. Uncontrolled, quench freezing causes ice crystals to form randomly, creating an isotropic network of interpenetrating pores (O'Brien et al., 2004). When such isotropic scaffolds are used, cells can infiltrate and grow in any direction. A controlled freezing rate controls the directionality of the pores within the scaffold by causing the ice crystals to align uniaxially, ultimately creating channel-like formations of porosity. Directionality of pores is facilitated by controlling the freezing rate; however, there are several methods to implement this principle such as using an insulative mold or by varying the freezing apparatus.

The most direct method of creating anisotropy within a scaffold is by controlling the cooling rate. In this method, the material is placed into a shelf freeze-dryer and the shelf-temperature of the freeze-dryer is decreased at a constant rate until it reaches the final temperature. This method generates pores with a more homogeneous size than scaffolds using an uncontrolled freezing process (O'Brien et al., 2004). Freezing rate can also be controlled by lowering the sample into a reservoir of liquid nitrogen at a specific rate; this method has been used to create polyvinyl alcohol (PVA) scaffolds (Zhang et al., 2005). Another method to induce the temperature gradient necessary to generate aligned pores is to load a biomaterial sample into an insulative mold, such as a polytetrafluoroethylene (PTFE) mold, and place on top of a cooled block. This creates a temperature gradient, as the PTFE mold insulates the material from the environmental temperature. In this case, the freezing front propagates from the bottom of the material, resulting in alignment of ice crystals as the material freezes (Asuncion et al., 2016). This method has been utilized to produce anisotropic silk fibroin scaffolds (Mandal and Kundu, 2009), PLLA scaffolds (Ko, 2020), and collagen-GAG scaffolds (Basurto et al., 2021). Characteristics of the molds used to induce directional freezing, such as the material type and the dimensions of the mold, have also been varied to measure their effects on the pore architecture of collagen scaffolds (Davidenko et al., 2012). In a study by Pot et al., anisotropic scaffolds were made using an aluminum/obomodulan wedge block to unidirectionally freeze collagen. The authors changed the pore size within these scaffolds by varying parameters such as freezing temperature and collagen concentration, while still maintaining pore alignment (Pot et al., 2015). Both of these freeze-drying methods (e.g., gradually decreasing the temperature of the freeze-dryer or keeping the temperature constant but insulating the scaffold material), create a temperature gradient which facilitates the formation of anisotropic pores within the material.

There are also methods of creating anisotropic alignment within scaffolds beyond the freeze-drying approach. One of these methods involves the use of magnetic particles as porogens. Before polymerization, these particles can be patterned in the presence of a magnetic field. Guo et al. fabricated a collagen scaffold with aligned pores by mixing iron oxide particles into a type I collagen solution (Guo and Kaufman, 2007). The solution was added to a microscope slide, and then a magnetic stir bar was added either underneath or on top of the slide. The samples along with the magnet were placed into a 37°C incubator until polymerized. This method resulted in the formation of collagen gels with aligned fibers, as the iron oxide particles within the collagen were able to pull the collagen fibers in the direction of the magnetic field (Guo and Kaufman, 2007). Another approach to induce fibrillar alignment of biomaterials is to physically strain the materials. Applying axial stretching to fibrin and collagen materials induced reorganization of fibrin fibrils within the materials (Pins et al., 1997; Brown et al., 2009; Grasman et al., 2014). This reorganization created more alignment upon these microthreads by inducing the formation of axially aligned topographical architecture (Grasman et al., 2014). These stretching methods are an effective method of inducing fibrillar alignment for use in regeneration of muscle fibers, and, importantly, represent an alternate approach to scaffold alignment without relying on pore formation.

## 4 Biomaterials used in skeletal muscle regeneration

Many biomaterials have been utilized for research in skeletal muscle tissue regeneration. The major classes of biomaterials that we will discuss in this review are natural, synthetic, and conducting polymers. While each of these biomaterial classes have shown promise in the field of skeletal muscle tissue engineering, they all have varying characteristics, advantages, and disadvantages. For instance, the mechanical, structural, and physicochemical properties of synthetic materials are more tunable than those of natural materials. Natural materials, however, contain bioactive cues, which synthetic materials lack (Qazi et al., 2015). Since these are all desirable characteristics, semi-synthetic composite biomaterials are created, in which natural and synthetic biomaterials are combined to obtain the desirable properties of each class. This can improve tissue engineering outcomes (Speidel et al., 2022). Conducting polymers are a specific subclass of synthetic polymers that are innately electrically conductive (Qazi et al., 2015). These material classes each have beneficial effects on skeletal muscle regeneration and will be elaborated upon in the subsequent sections of this review.

### 4.1 Natural materials for skeletal muscle regeneration

In this section, we will review the most common types of natural biomaterials. Several types have been studied for use in skeletal muscle engineering applications. Some examples of these materials are summarized and defined in Table 1, as well as the advantages and limitations of these materials in the context of skeletal muscle regeneration.

**TABLE 1:** Advantages and limitations of natural biomaterials for skeletal muscle regeneration.

Material	Definition	Advantages	Limitations	References
Collagen	Structural protein found in the ECM throughout the body	Facilitates myotube formation, supports revascularization	Mechanical strength	Kin et al. (2007), Kinneberg et al. (2010), Qazi et al. (2015)
Gelatin	Denatured form of collagen	Superior mechanical properties, slow degradation rate	Did not recruit SCs *in vivo*	Gattazzo et al. (2018), Haas et al. (2019), Gupta et al. (2021)
Alginate	Naturally occurring polysaccharide found in seaweed	Sustained release of growth factors, modulates immune response	Cells express lower levels of myogenic genes when seeded on alginate scaffold	Baniasadi et al. (2016), Yi et al. (2017), Pollot et al. (2018)
Chitosan	Polysaccharide harvested from crustaceans	Antimicrobial properties	Mechanical strength may be too high	Jana et al. (2013), Kheradmandi et al. (2016), Tonda-Turo et al. (2017)
Fibrin	ECM protein involved in the coagulation cascade for hemostasis	Induce high levels of skeletal muscle gene expression	Rapid degradation rate	Huang et al. (2005), Fischer et al. (2021), Genovese et al. (2021)
dECM	Material remaining after the cellular components of a tissue have been removed	Biocompatibility and structural support	Lack of precision in manufacturing	Sicari et al. (2014), Sarrafian et al. (2018), Urciuolo and De Coppi (2018)

Collagen as a biomaterial has low antigenicity, good biodegradability, and biocompatibility (Rezvani Ghomi et al., 2021). It also has tunable mechanical properties, which can be optimized during fabrication as well as with post-fabrication processes, such as crosslinking, and promotes cell growth because of the presence of several cell binding motifs throughout its triple helical structure (O'Brien et al., 2005; Qazi et al., 2015). Various methods have been employed to fabricate aligned collagen scaffolds, such as electrospinning, directional freeze-drying, cyclic stretching, magnetic alignment, extrusion, and microfluidics (Dewle et al., 2020). In a study by Kroehne et al., type I collagen scaffolds with aligned pore structure were seeded with C2C12 myoblasts, which formed myotubes and aligned parallel to the pore alignment. In addition to robust differentiation, these myotubes secreted their own ECM as revealed by immunostaining against laminin (Kroehne et al., 2008). The *tibialis anterior* (TA) and *extensor digitalis longus* (EDL) muscles of mice were removed, and C2C12-seeded sponges were implanted into the injury site. This scaffold supported the regeneration of both muscle and tendon and facilitated tissue revascularization (Kroehne et al., 2008). Porous collagen scaffolds have also been shown to induce vascularization (Ma et al., 2011) as well as facilitate cell ingrowth and alignment and subsequent integration with the surrounding tissue after implantation (Brouwer et al., 2013). The regenerated tissue in the study by Kroehne et al., however, did not have the same mechanical strength as uninjured muscle, and the resulting forces were 5%–20% of the uninjured force values (Kroehne et al., 2008). These results show that full restoration of force production to pre-injured or healthy muscle values has not yet been achieved, highlighting the need for improved tissue engineering strategies.

Gelatin is commonly utilized for the delivery of cells into a specific defect area and for drug delivery applications (Fischer et al., 2021). Gelatin has also been shown to facilitate cell adhesion and proliferation, in addition to being biodegradable and non-immunogenic (Acevedo et al., 2019). Gelatin scaffolds can be fabricated with a wide stiffness range (2–75 kPa) based on the concentration of gelatin used. Gelatin scaffolds can thus be tuned to have a stiffness value which mimics that of the elastic modulus of skeletal muscle (∼12–16 kPa) (Jana et al., 2016; Chaturvedi et al., 2017), making it attractive for use in skeletal muscle regeneration (Gattazzo et al., 2018). Aligned gelatin scaffolds are mainly produced through electrospinning, however other methods of aligned scaffold fabrication such as unidirectional freeze-drying (Wu et al., 2010), 3D printing (Tijore et al., 2018), and centrifugal spinning (Loordhuswamy et al., 2014) have been employed. Porous gelatin scaffolds have also been shown to induce satellite cell infiltration and vascularization, both contributing to skeletal muscle regeneration (Ju et al., 2014). Gelatin concentration can also affect the microstructure of the scaffold and thus the behavior of the seeded cells. Scaffolds made from 7.5% *w/v* gelatin were directionally frozen to create aligned pores, and they supported a uniform distribution of aligned myoblasts throughout the material. While 7.5% *w/v* gelatin scaffolds resulted in a structure more like that of connective muscle tissue, scaffolds made from lower concentrations of gelatin (2.5% and 5% *w/v*) exhibited a lamellar-like structure, and scaffolds made from higher concentrations (10% and 12.5% *w/v*) exhibited irregular tubular structures (Hu et al., 2022). In a study conducted by Haas et al., the authors fabricated composite scaffolds with a gelatin to collagen ratio of 100:0 (only gelatin), 90:10, or 70:30. Scaffolds containing a higher ratio of gelatin (90:10) had a higher compressive modulus and a larger average pore size than scaffolds with a lower gelatin ratio (70:30). Scaffolds made with higher gelatin concentrations (100:0 or 90:10) had higher peak load and peak stress values than the 70:30 scaffolds. Despite this improvement in mechanical properties, composite sponges with higher gelatin concentrations supported less cell proliferation than sponges with higher concentrations of collagen (Haas et al., 2019). The use of gelatin in biomaterial scaffolds may also be somewhat limited by its high sensitivity to enzymatic degradation (Afewerki et al., 2019) and lack of thermostability (Lukin et al., 2022), although these concerns can be addressed through crosslinking (Gupta et al., 2021).

Fibrin acts as the provisional wound healing matrix which facilitates tissue repair and has been utilized as a biomaterial. In a study exploring the formation of ECM by smooth muscle cells (SMCs) within a fibrin gel construct, SMCs were reported to fabricate their own ECM to replace the degrading fibrin in as soon as 2–4 weeks (Ross and Tranquillo, 2003; Marcinczyk et al., 2017). Fibrin can be used for growth factor delivery and combined with other proteins to make a more bioactive scaffold, as it has several heparin-binding domains and multiple binding sites for growth factors and bioactive ECM molecules, such as fibronectin (Marcinczyk et al., 2017). Fibrin has been fabricated into hydrogels (Huang et al., 2005; Marcinczyk et al., 2017; Fischer et al., 2021; Genovese et al., 2021; Ziemkiewicz et al., 2022), microthreads (Page et al., 2011; Grasman et al., 2012; Grasman et al., 2015a; Grasman et al., 2017; Carnes and Pins, 2020a), and electrospun matrices (Gilbert-Honick et al., 2018a; Gilbert-Honick et al., 2018b) for use in skeletal muscle regeneration. Porous, electrospun fibrin scaffolds with aligned fiber topography were implanted into a TA VML defect model in mice, either with or without pre-seeding with C2C12s (Gilbert-Honick et al., 2018b). Functional recovery was assessed by measuring the maximum isometric torque of the treated muscle at 2 and 4 weeks post-implantation, and treatment with either cellular or acellular scaffolds exhibited maximum isometric torque values equal to those of uninjured controls (Gilbert-Honick et al., 2018b). Fibrin microthreads are an anisotropic cylindrical scaffold along which myoblasts can proliferate and differentiate into myofibers (Grasman et al., 2017). These scaffolds have been implanted into a mouse TA model of VML either pre-seeded with myoblasts and (Page et al., 2011) or loaded with growth factors to encourage endogenous skeletal muscle repair (Grasman et al., 2015a). Both of these studies support the use of fibrin-based materials for skeletal muscle repair, as numerous new myofibers formed in the wound site after implantation, resulting in significant functional improvements. The application of mechanical strain to cell-seeded fibrin scaffolds prior to implantation makes these scaffolds even more effective at inducing aligned myofiber formation (Heher et al., 2015). Fibrin is therefore a promising biomaterial for use in regenerating VML injuries.

Decellularized extracellular matrix (dECM) has shown promise as a scaffold for skeletal muscle regeneration. dECM is porous and aligned based on the orientation and geometry of the original tissue. The preparation of these scaffolds is predominately done with standardized protocols, leading to wide use and commercial availability. Sources for dECM can vary, such as small intestinal submucosa (SIS), urinary bladder matrix (UBM), dermis, or various skeletal muscles. SIS and UBM scaffolds specifically have been clinically approved and shown success in use within humans as well. The sources of the ECM may cause the resulting scaffolds to have varying characteristics; for example, SIS and UBM scaffolds are similarly effective, while dermal ECM has better mechanical stability and higher potential to induce myogenesis *in vitro* than UBM scaffolds (Wolf et al., 2012; Sarrafian et al., 2018). These ECM-based scaffolds are beneficial because they are biocompatible, and upon degradation they can release chemoattractant and antimicrobial peptides, as well as growth factors and extracellular vesicles which help to attract stem cells. dECM scaffolds can induce an immune response which is beneficial for constructive remodeling of the tissue (Mase et al., 2010; Sicari et al., 2012; Dziki et al., 2017a; Dziki et al., 2017b). Research has demonstrated that porous dECM scaffolds can facilitate cell growth, alignment, and myotube formation (Smoak et al., 2021). Porous dECM scaffolds also promote vasculature formation and can integrate with the host tissue (Hogan et al., 2022). A study was conducted with patients who had experienced VML, resulting in three out of five of them regaining muscular function after the addition of a SIS hydrogel scaffold (Sicari et al., 2014). A 13-patient study showed that after 6 months, patients who received implantation of a dECM scaffold as a treatment for VML had an average of 37.3% improvement in strength as compared to pre-operative values (Dziki et al., 2016). Unfortunately, dECM scaffolds present some disadvantages as well. The manufacturing process of dECM is much less precise compared to polymeric biomaterials, leading to variability in the shape, mechanical properties, and structural properties of the material. Beyond this, the manufacturing of various dECM skeletal muscle scaffolds can be much more difficult due to the complexity of the tissue used (Urciuolo and De Coppi, 2018).

Alginate is also biocompatible, has low toxicity, and has been shown to restrain the maturation of dendritic cells, which can diminish an inflammatory immune response to the material (Baniasadi et al., 2016). Methods such as unidirectional freezing (Francis et al., 2013; Almeida et al., 2017), fiber extrusion methods (Kang et al., 2012), microfluidics (Lee et al., 2009), and electrospinning (Tonsomboon and Oyen, 2013) have been used to induce alignment in alginate-based scaffolds. Aligned alginate scaffolds have been shown to support C2C12 cell viability (Yeo and Kim, 2018), as well as myogenic differentiation (Yeo and Kim, 2019). In a study by Pumberger et al., alginate solution was mixed with IGF-1 and vascular endothelial growth factor (VEGF), frozen, and lyophilized to form porous, isotropic scaffolds (Pumberger et al., 2016). The scaffolds were transplanted into the left soleus muscle of rats, which had undergone a blunt crush trauma. Results showed that these alginate materials were able to provide sustained release of the growth factors to an injury site and, in combination with pre-seeding with mesenchymal stromal cells, resulted in improved fast twitch muscle force 56 days post-transplantation when compared to alginate scaffolds lacking the growth factors and cells. These porous scaffolds also increased muscle fiber density and vascularization in the wound site (Pumberger et al., 2016). Separately, an injectable form of alginate was used to deliver VEGF, IGF-1, and myoblasts to a muscle injury, which facilitated an increase in weight of the recovered muscle, blood vessel density, and force output (Borselli et al., 2011). Cells seeded on alginate scaffolds expressed higher levels of desmin and myosin than cells seeded on scaffolds made of other materials such as Matrigel, suggesting that scaffolds made from alginate can support and enhance all stages of myoblast differentiation (Yi et al., 2017). Despite the clear ability to load and deliver varying growth factors, supporting *in vivo* regeneration of injured skeletal muscle, and enhancing *in vitro* culture and differentiation of myoblasts, some drawbacks still exist. An undesirable characteristic of alginate is that non-modified alginate is non-degradable and can cause a foreign body response (Balakrishnan et al., 2014). Alginate also does not contain cell-binding motifs, potentially limiting its biocompatibility and ability to direct cell functions (Datta et al., 2018).

Chitosan is most commonly used as a wound dressing for hemostatic stabilization, and has been used as a scaffold because of its intrinsic antimicrobial properties (Jana et al., 2013). Chitosan is beneficial because it exhibits antibacterial, antifungal, and antitumorigenic properties, and it has also been shown to reduce clotting time (de Sousa Victor et al., 2020). Porous and aligned chitosan sponges have been produced through freeze drying (Madihally and Matthew, 1999; Jana et al., 2013) and electrospinning (Tonda-Turo et al., 2017). In a study by Jana et al., chitosan was freeze-dried to form porous scaffolds with aligned pores, which were approximately 50 µm in diameter (Jana et al., 2013). Changing the chitosan concentration altered the resulting pore size: increasing the chitosan concentration caused the pore size to decrease. C2C12 myoblasts were seeded on these scaffolds to assess myotube formation, and myotubes on the scaffolds with the highest chitosan concentration had the largest diameter (Jana et al., 2013). Tonda-Turo et al. electrospun chitosan to produce aligned, porous scaffolds. C2C12s that were seeded on these aligned scaffolds showed favorable viability and enhanced myofiber alignment and elongation (Tonda-Turo et al., 2017). In a study determining the effects of modifying the ratio of alginate:chitosan in a freeze-dried scaffold, it was shown that a pure chitosan scaffold had limited cell retention capabilities as compared to scaffolds made with a mix of alginate and chitosan; cell number on these scaffolds was shown to decrease over a 2-week period (Bushkalova et al., 2019). While chitosan has shown promising results in *in vitro* studies, potential limitations of chitosan are that it has low solubility and a lack of long-term stability, which may impact its ability to direct functional regeneration after VML injury (Drewnowska et al., 2013).

### 4.2 Synthetic polymers for skeletal muscle regeneration

Synthetic polymers have become key materials of study in the search for an effective and compatible scaffold for muscle regeneration after VML. The lack of complete control over chemical moiety and protein sequence, along with the more rapid degradation rate of natural materials are some limitations of these materials (Grasman et al., 2015b), which have spurred further investigation into synthetic polymers as biomaterial scaffolds for skeletal muscle tissue engineering. Synthetic polymers including polyurethane, poly-l-lactic acid (PLLA), PCL, and their copolymers are common alternatives to organically derived scaffolds (Table 2) (Guo et al., 2013). These are aliphatic polyesters and possess useful qualities such as biocompatibility, biodegradability, suitable mechanical properties, and non-toxicity (Place et al., 2009; Guo et al., 2013). In addition to acting as scaffolds themselves, these can be combined with conductive polymers and biological materials to create composite materials which can possess the mechanical, conductive, and biocompatible properties necessary to regenerate skeletal muscle tissue.

**TABLE 2:** Advantages and limitations of synthetic biomaterials for skeletal muscle regeneration.

Synthetic polymers	Advantages	Disadvantages	Citations
PCL	High Young’s modulus, stiffness	Does not support cell proliferation or differentiation alone	Sundelacruz and Kaplan (2009), Kim et al. (2010), Sánchez-Cid et al. (2021)
	New muscle growth when coated with natural biomaterials	Long degradation times	
PLGA	Mild support of differentiation	Lower elastic modulus when combined with natural biomaterials	Boateng et al. (2005), Aviss et al. (2010), Shin et al. (2015), Wang et al. (2021)
	Good differentiation when coated with natural materials	Acidic degradation products	
PEG	Hydrophilic	Does not support proliferation or differentiation alone	Fuoco et al. (2015), Kutikov and Song (2015), Wang et al. (2019)
	New muscle growth when coated with biomaterials	Requires functionalization to degrade	
	Addition of PEG has plasticizing effect on hard polymers		
PLLA	Grows functional muscle in combination with other biomaterials	More successful with protein coating	Scime et al. (2009), Lee et al. (2012), Wolf et al. (2015), Fitzgerald et al. (2018)
	Recruits native SCs	Acidic degradation products	
	Supports vascularization	Long degradation times	
PGA	Hydrophilic	Does not induce vascularization	Saxena et al. (2001), Fuchs et al. (2003), Kamelger et al. (2004), Miranda et al. (2021)
		Rapid degradation	
		Acidic degradation products	
PDMS	Grows 2D skeletal muscle films	Ineffective without combining with other natural materials	Fujita et al. (2009), Shen et al. (2013), Mueller et al. (2021)
	Useful as a mold or patterned surface	Little *in vivo* research	
		Does not degrade *in vivo*	

PCL is a synthetic aliphatic, biodegradable polymer (Nevoralová et al., 2020). In a study by Qian et al., porous PCL scaffolds were made using microneedles. These scaffolds were implanted into the sciatic nerve of rats to act as a nerve conduit and facilitated the growth of larger muscle fibers than untreated nerve injuries (Qian et al., 2020). Electrospun, porous PCL scaffolds, supplemented with 5-azacytidine, have been shown to support the differentiation of human mesenchymal stem cells into mature myofibers (Fasolino et al., 2017). Porous PCL scaffolds which were coated with a nitrogen-functionalized hydrocarbon coating supported the formation of myofibers using C2C12 myoblasts (Giraud et al., 2007). Fused deposition modeling has been used to create scaffolds with aligned PCL fibers (Zein et al., 2002), as well as electrospinning (Koepsell et al., 2011). C2C12s seeded on an aligned PCL scaffold showed elongated morphology, while C2C12s seeded on a non-aligned PCL scaffold showed circular morphology. The elongated cells differentiated to form aligned myotubes, while the cells on the non-aligned scaffold formed randomly oriented myotubes (Yang et al., 2019). PCL, however, is hydrophobic, and it has been shown that pure PCL (*i.e.,* with no additions or coatings) does not sufficiently support skeletal muscle proliferation or differentiation (Sundelacruz and Kaplan, 2009; Kim et al., 2010; Woodruff and Hutmacher, 2010; Qazi et al., 2015; Perez-Puyana et al., 2021). Therefore, proteins and natural polymers such as gelatin and collagen have been attached to its surface to improve cell adhesion and compatibility (Sundelacruz and Kaplan, 2009; Woodruff and Hutmacher, 2010). The addition of gelatin significantly increased myogenic gene expression (Kim et al., 2010; Perez-Puyana et al., 2021); however, the mechanical properties of these composite materials were generally reduced compared to pure PCL, with both a lower Young’s modulus and lower ultimate tensile strength (Perez-Puyana et al., 2021). The addition of 2% elastin to PCL scaffolds increased the Young’s modulus and maximum stress, but further increases in elastin concentration led to a sharp deterioration in these mechanical properties (Sánchez-Cid et al., 2021). Muscle dECM incorporated into porous PCL, which was fabricated through salt leaching, induced *de novo* muscle growth, did not induce an inflammatory response, and resulted in a higher MHC-collagen ratio when implanted into a murine VML model (Lu et al., 2009; Jin et al., 2021b). Therefore, PCL-biological mixtures have high potential to reduce the formation of fibrous scar tissue and support muscle growth in injury sites. PCL has some limitations, however, in that it is hydrophobic and has low wettability, leading to poor cell attachment. The solvents used with PCL are also somewhat toxic (Ilyas et al., 2022). Additionally, PCL has a very slow degradation rate; it can take 2 years for this material to fully degrade (Arif et al., 2022), and it seems to be most promising as a biomaterial for skeletal muscle tissue engineering when natural materials are combined with it, suggesting that it may not be an ideal choice to repair this tissue.

PLLA is a synthetic polymer which has shown promise as a biomaterial due to its versatility for use in various tissue types, namely because its characteristics such as porosity, rigidity, and degradability can be tuned for various purposes. It has often been fabricated into aligned scaffolds through electrospinning (Dai et al., 2022). Aligned poly lactic acid scaffolds, produced by electrospinning, were cultured with C2C12 cells and motor neurons, and it was found that mature, densely packed myofibers formed on these scaffolds. In a long-term co-culture of these 2 cell types, it was found that these scaffolds facilitated the formation of neuromuscular junctions (Luo et al., 2018). Porous, PLLA-based scaffolds have also shown some promise in the ability to recruit native SCs to regrow muscle tissue when implanted *in vivo* (Lee et al., 2012; Ju et al., 2014; Wolf et al., 2015). The degradation rate of PLLA, however, is relatively slow (Fitzgerald et al., 2018). Additionally, microfibrous polylactic acid scaffolds have exhibited promising results in directing myofiber orientation in a uniform direction, but remain limited by their high tensile modulus (in the range of GPa), which can negatively affect skeletal muscle regeneration (Riboldi et al., 2005). PLLA has thus been combined with other materials to form more effective scaffolds for skeletal muscle regeneration. PLLA scaffolds incorporating gelatin were more successful than virgin PLLA scaffolds because the addition of gelatin aided myoblast attachment (Cronin et al., 2004). Additionally, porous PLLA scaffolds, fabricated into sponge-like materials, show potential to address revascularization, a major challenge for skeletal muscle regeneration. PLLA scaffolds have been seeded with myoblasts and endothelial cells to increase vascularization after implantation (Levenberg et al., 2005; Landau et al., 2017). PLLA fibers, when coated with ECM proteins such as laminin or fibronectin, have been shown to increase the growth and differentiation of skeletal muscle myoblasts into multinucleated myofibers, combining the fibrous architecture of the PLLA scaffold to direct parallel growth of myofibers and the biological signals of the dECM to grow functional muscle (Cronin et al., 2004; Scime et al., 2009). PLLA is a polymer with much precedent for growing skeletal muscle *in vivo* but may be more successful with the incorporation of natural biomaterials.

Polyglycolic acid (PGA) is an aliphatic polyester that is hydrophilic and biodegradable, but also possesses high mechanical strength (Niaounakis, 2015). It can be fabricated into porous scaffolds which degrade over time (Qazi et al., 2015). The major method of producing aligned PGA scaffolds is through electrospinning (Boland et al., 2001; Barnes et al., 2007). A study by Kamelger et al. showed that myoblast-seeded PGA constructs supported vascularization and myotube formation (Kamelger et al., 2004; Qazi et al., 2015). Research by Pedrotty et al. shows PGA meshes supported the proliferation of myoblasts, but did not greatly affect their differentiation (Pedrotty et al., 2005; Nakayama et al., 2019). Other studies show that PGA meshes seeded with myoblasts have induced the formation of well-vascularized structures and tissue very similar to new muscle when implanted into the omentum in the peritoneal cavity (Saxena et al., 2001; Scime et al., 2009). This may be because acellular PGA cannot induce host myofiber formation; pre-seeding the scaffold with myogenic cells may be useful for facilitating new tissue formation. Another potential concern with PGA is its rapid degradation *in vivo*—this timeframe is usually only a few weeks (Kamelger et al., 2004; Niaounakis, 2015; Nakayama et al., 2019). Zhang et al. quantified the *in vivo* degradation of porous PGA foams using fluorescence intensity. Results showed that the fluorescence intensity of PGA foams dropped to 20% of their original intensity after 3 weeks, indicating rapid degradation. Additionally, this rapid degradation created an acidic environment, which recruited M1 macrophages to the implantation site, thus prolonging inflammation (Zhang et al., 2020). PGA scaffolds have the potential to develop vascularized and aligned new muscle tissue but should be combined with a more slowly degrading material, such as PCL or PLA, to produce optimal results (Kumar et al., 2021).

PLGA is a biocompatible and biodegradable synthetic polymer that has been used in biomedical applications for decades (Makadia and Siegel, 2011). This copolymer is composed of PLLA and PGA, and changing the final ratio of these monomers can impact the final material properties. For example, solubility of PLGA and its melting temperature decreases with increasing PGA content, and its degradation rate decreases as the PLLA:PGA ratio increases (Jin et al., 2021a). Aligned PLGA scaffolds can be produced through electrospinning; in a study comparing aligned and non-aligned PLGA scaffolds and their effect on PC12 cells, it was found that the PC12 cells formed more clusters and exhibited a more elongated morphology when seeded upon aligned scaffolds, suggesting the ability to support aligned growth along the scaffold (Mehrasa et al., 2015). Aviss et al. created aligned, porous PLGA scaffolds through electrospinning, and reported that 13% more myotubes formed on pure PLGA scaffolds over 14 days, which was significantly higher than the control (Aviss et al., 2010). In a study by Levy-Mishali et al., porous scaffolds were made from pure PLLA, pure PLGA, or PLLA and PLGA at ratios of 75/25, 50/50, or 25/75 PLLA/PLGA. Porosity was achieved using salt leaching. Interestingly, pure PLGA scaffolds, when cultured with muscle myoblasts, supported the lowest levels of cell viability as well as the largest levels of shrinkage, as caused by cell contraction (Levy-Mishali et al., 2009). To further improve cell adhesion or function, PLGA scaffolds can be functionalized with peptide sequences, such as RGD. The functionalization of PLGA with RGD enhanced the growth and differentiation of C2C12 myoblasts (Wang et al., 2013). Electrospun, porous PLGA has also been combined with dECM, which increased myotube width, multinucleation, and formation as compared to pure PLGA materials (Aviss et al., 2010). However, dECM-PLGA composite scaffolds had a much lower Young’s modulus than the pure PLGA scaffolds, showing a decrease in mechanical properties when biological materials are added. PLGA has also been fabricated into microspheres for drug delivery and regenerative medicine (Bae et al., 2009). This idea has recently been used in VML therapy investigation: Wang et al. found that injection of PLGA microspheres with polyethylene glycol (PEG) microrods into a murine VML model improved *in situ* muscle regeneration, as demonstrated by proliferation of myoblasts at the injection site (Wang et al., 2021). PLGA thus has positive results when combined with natural materials. However, the mechanical properties of these composite materials are often lacking. Additionally, PLGA materials may produce acidic byproducts upon degradation, which can result in inflammation (Ko et al., 2021).

PEG is a synthetic polyether that is unlike many other synthetic biomaterials because of its hydrophilicity, as most are generally hydrophobic (Wang et al., 2019). As a scaffold biomaterial, PEG has exhibited non-toxicity, good biocompatibility, and low immunogenicity (Kong et al., 2017). PEG hydrogels can be formed with aligned architecture using molds and photolithographic patterning techniques (Zhang et al., 2015). PEG has also been chemically tethered to other biomaterials and unidirectionally frozen (Yang et al., 2020) or electrospun (Jiang et al., 2015; Karahaliloğlu, 2017) to form aligned scaffolds. However, its lack of biological signaling capability means that the incorporation of biological proteins or peptide sequences is necessary. Dong et al. found that myotubes did not form on PEG films when they were seeded with myoblasts, showing that PEG does not support myoblast differentiation (Dong et al., 2017). Different methods have been employed to mitigate this lack of bioactivity, such as coating the porous PEG scaffold in collagen to induce the attachment of skeletal myoblasts (LaNasa et al., 2011), or adding peptide sequences such as YRGDS to the scaffold to facilitate regeneration (Chiu et al., 2011). PEG scaffolds functionalized with RGD were fabricated into hydrogels containing aligned channels. These scaffolds were able to support a high density of skeletal muscle myoblasts, which formed three-dimensional alignment and ultimately formed myotubes (Hume et al., 2012). PEG hydrogels containing fibrinogen were able to support the growth and differentiation of C2C12 myoblasts, but the myotubes produced were thinner than those grown on collagen and fibrin hydrogels. These findings suggest that there is a reduced ability of myoblasts to adhere to synthetic matrices (Fuoco et al., 2015; Prüller et al., 2018). Studies therefore reveal the use of PEG in biomaterial scaffolds is limited by its lack of ability to support skeletal muscle cell adhesion.

Polydimethylsiloxane (PDMS) is a hydrophobic elastomer with good optical, electrical, and mechanical properties as well as biocompatibility (Ionescu et al., 2012; Miranda et al., 2021). PDMS can be fabricated into porous scaffolds by pouring PDMS into 3D printed molds, and by modulating the polymerization process the resulting Young’s modulus of the scaffolds can be tuned to a wide range of values (52–1,038 kPa) (Montazerian et al., 2019). Another common use for PDMS in skeletal muscle tissue engineering is as a micro-grooving stamp for biological hydrogels, especially gelatin-based, resulting in the formation of long, well-aligned myotubes of a uniform diameter (Hosseini et al., 2012; Bettadapur et al., 2016; Ostrovidov et al., 2017; Alheib et al., 2021). PDMS has been used effectively for the growth of 2D skeletal muscle films (Shen et al., 2013; Alarcin et al., 2021). Aligned topography can be formed in PDMS through micropatterning (Lam et al., 2006; Huang et al., 2010). Patterned pure PDMS surfaces, which were seeded with myoblasts and endothelial cells, resulted in myofiber formation and production of ECM components such as collagen and laminin, suggesting that PDMS supports the growth of new muscle tissue. Increased laminin production was found on PDMS surfaces with directional topography, as opposed to flat PDMS (Almonacid Suarez et al., 2020). Coating PDMS with materials such as PGA, collagen, and laminin enhances its ability to support muscle tissue growth, which can generate active tension upon electrical stimulation (Fujita et al., 2009; Mueller et al., 2021). To our knowledge, however, PDMS has been used mainly *in vitro* for skeletal muscle regeneration applications and not *in vivo*. PDMS is mainly as a template material for the fabrication of scaffolds or for cell culture and *in vitro* myofiber formation (Bian and Bursac, 2009; Huang et al., 2010).

### 4.3 Conductive polymers for skeletal muscle regeneration

Skeletal muscle functionality relies on electrical stimulation, typically in the form of action potentials from neurons across the NMJ, for contraction and force propagation. Therefore, conductive materials are especially important in the construction of a skeletal muscle tissue engineering scaffold, as there is increasing evidence that additional stimulation can assist in muscle maturation (Langelaan et al., 2011). To fabricate conductive polymers (CPs), polymers must undergo a “doping” process, which introduces charge into the polymer and causes it to be conductive. P-doping is the process of oxidizing a polymer and thus giving it a positive charge, while n-doping is the process of reducing a polymer and thus giving it a negative charge. Doping can be conducted chemically, electrochemically, or though photodoping (Balint et al., 2014). The dopant molecule used can also affect the characteristics of the polymer—for example, larger dopants can greatly affect the material properties (such as density) of the polymer, and can provide the polymer with greater electrochemical stability since they do not leach out of the polymer over time (Balint et al., 2014). CPs have shown low toxicity and promising results with tissue engineering projects in tissues that respond to electrical stimulation, including nerve, skeletal muscle, cardiac muscle, bone, fibroblasts, and others (Ateh et al., 2006a; Ateh et al., 2006b; Jeong et al., 2008; Quigley et al., 2009; Ghasemi-Mobarakeh et al., 2011). Yet, the use of CPs poses several challenges, including poor solubility in organic solvents, insufficient interaction with cells, and brittleness, which may be caused by the doping process used (Green et al., 2008; Place et al., 2009; Guo et al., 2013; Guo and Ma, 2018; Dong et al., 2020). Another issue with CPs is their non-biodegradability; extended use of CP-containing scaffolds *in vivo* has resulted in inflammation and a second procedure has been required to remove the scaffold (Guo et al., 2013). However, when combined with copolymers and biomaterials in the form of composites, CPs have shown beneficial results that indicate potential for skeletal tissue engineering (Table 3). In fact, due to their brittleness and non-biodegradability, CPs have almost entirely used by adding the CP into another biomaterial.

**TABLE 3:** Advantages/disadvantages of conductive polymers used for skeletal muscle regeneration.

Conductive polymer	Advantages	Disadvantages	Citations
Polyaniline (PANi)	Better differentiation of tissue when added to copolymers Improved differentiation More and longer myotubes in PANi-containing scaffolds	Slightly cytotoxic No effect on cell adhesion and proliferation Brittle	Green et al. (2008), Kim et al. (2009a), Liu et al. (2010), Guo and Ma (2018)
Polypyrrole	Supports cell proliferation and differentiation Increases conductivity of scaffold without decreasing metabolic activity of cells	Low strength Brittle	Green et al. (2008), Guo and Ma (2018), Basurto et al. (2021), Bilge et al. (2021)
Polythiophene	Easily modifiable Soluble in organic solvents	Addition of polythiophene reduces Young’s modulus Low elasticity Brittle	Green et al. (2008), Jaymand et al. (2016), Guo and Ma (2018), Massoumi et al. (2021)

Polyaniline (PANi) is a conductive polymer that is widely used in biomedical applications because of its biocompatibility, low cytotoxicity, low cost, and ease of synthesis (Humpolicek et al., 2012; Qazi et al., 2014). PANi is often used in conjunction with other biocompatible materials, including gelatin, collagen, silk fibroin, PCL, PLLA, and PLGA, particularly for the formation of 3D structures including bioactive scaffolds (Arteshi et al., 2018). The addition of PANi to aligned, porous PCL scaffolds, produced by electrospinning, improved C2C12 proliferation and differentiation (Chen et al., 2013). Srisuk et al. fabricated porous, sponge-like gellan gum (GG) scaffolds with and without PANi, and found that C2C12 myoblasts exhibited an elongated morphology on the PANi-GG scaffolds within 24 h, while it took 48 h for myoblasts to elongate on the pure GG scaffolds. After 7 days, myotubes formed on the PANi-GG scaffolds, but not on the GG scaffolds (Srisuk et al., 2018). These findings indicate that PANi is an effective material to use for skeletal muscle cells, however, myoblasts exhibited a lower rate of growth on conductive PANi films during the first 100 h of culture. Acids such as hydrochloric acid (HCl) and sulfonic acid derivatives are the most used dopants for PANi. Bidez III et al. found that using HCl to dope PANi may leave behind small amounts of acid dopants within the material that leak from the material over time, thus having a negative impact on cell viability and proliferation (Bidez et al., 2006). The cytotoxicity caused by PANi films could be detrimental to the growth of skeletal muscle tissue *in vitro*. There were no significant differences in the growth rates of porcine skeletal muscle myoblasts when cultured on pure collagen or PANi-collagen composite materials (Kim et al., 2009a). Interestingly, when PANi-based scaffolds are designed to have an aligned architecture, they have a beneficial effect on the behavior of skeletal muscle cells. Ku et al. found that more myotubes were present on aligned scaffolds, which were fabricated through electrospinning and thus made to be porous, with PANi; additionally, expression of myogenin, troponin-T, and MHC were significantly higher than on scaffolds without PANi (Ku et al., 2012). Higher concentrations of PANi grafted onto PCL scaffolds also resulted in longer myotubes. Synthetic composites containing PANi have also been able to produce mature aligned and long myotubes (Ku et al., 2012). The ability of PANi-doped scaffolds to support aligned myotubes, in addition to higher expression levels of differentiation markers shows that PANi can aid in skeletal muscle development. However, these positive attributes of PANi are somewhat tempered by its cytotoxicity because of the acidic doping process, so its overall impact on skeletal muscle tissue engineering is unclear.

Polypyrrole (PPy) is a highly conductive polymer that is a focus of research in several tissue engineering applications (Liang and Goh, 2020). To our knowledge, it seems that pure PPy has not been fabricated into a porous structure, but mainly into films. PPy films aid in myoblast proliferation and differentiation: when combined with dopants in the form of films, every film was able to support the adhesion and proliferation of C2C12 myoblasts (Gilmore et al., 2009). PPy can be doped with biomolecules such as hyaluronic acid (HA), para-toluenesulfonic acid (pTS), dextran sulfate (DS), poly (2-methoxyaniline-5-sulfonic acid) (PMAS), and chondroitin sulfate (CS). It was found that PPy/PMAS and PPy/CS scaffolds supported skeletal muscle cell differentiation, while PPy/HA and PPy/pTS scaffolds were not effective at supporting skeletal muscle cell differentiation (Gelmi et al., 2010). Gilmore et al. found that on lower-roughness PPy films, myoblasts were able to both proliferate and differentiate into myofibers (Gilmore et al., 2009; Tallawi et al., 2016). This implies that PPy, unlike other conductive polymers studied, does not have significant cytotoxic effects and is able to support skeletal muscle differentiation on its own without needing to be combined with another polymer or biomaterial. However, PPy is also often used as a part of 3D structures such as scaffolds and hydrogels alongside copolymers and biomaterials to great effect. Aligned, porous PCL/PPy scaffolds supported the attachment, proliferation, and differentiation of C2C12 myoblasts (Browe and Freeman, 2019). When PPy nanoparticles were integrated into a porous collagen scaffold, the resulting structure had 5x the conductivity of a pure collagen scaffold without interfering with the metabolic activity of C2C12 myoblasts (Basurto et al., 2021). Basurto et al. found that PPy/collagen scaffolds also had increased MHC staining and more multi-nucleated myotubes as compared to pure collagen (Basurto et al., 2021). These findings indicate that PPy can greatly enhance myoblast differentiation and maturation *in vitro*, making it a highly promising conductive polymer for skeletal muscle tissue engineering. Multiple studies support these findings and suggest that PPy, when used as a biomaterial coating on scaffolds and in hydrogels, promotes myoblast adhesion, spread, and differentiation. However, while it reduces the tensile strength of the biomaterial, it does not seem to impact material stiffness (Berti et al., 2017; Browe and Freeman, 2019; Bilge et al., 2021; Zarei et al., 2021). PPy is thus a promising material due to its favorable effects on muscle cells. However, its negative impact on tensile strength as well as its brittleness may require it to be combined with other materials to effectively regenerate skeletal muscle.

Polythiophenes (PThs) are another group of conducting polymers of increasing importance in biomedical applications. PTh possesses an advantage over other conducting polymers because of its solubility in organic solvents, allowing it to be used with a broader range of materials, as well as its amenability to chemical modifications through add-on functional groups (Roncali, 1992; Collis et al., 2003; Kim et al., 2006). Much like many other CPs, PTh appears to be non-toxic and is largely biocompatible (Guo et al., 2013). PTh also supports the differentiation of skeletal muscle myoblasts through the increased development of myotubes and overall increases in the fusion index, with around 60%–65% of cells forming multinucleated myotubes after 3 days of culture on pure PTh films (Kim et al., 2006). Electrospinning was also used to make aligned, PTh-based fiber mats, which supported the *in vitro* alignment of muscle fibers (Breukers et al., 2010). Although PThs have been used in scaffolds and porous structures, their study with skeletal myoblasts has been limited; this may be due to its Young’s modulus being relatively higher than that of skeletal muscle, which conflicts with the requirements for skeletal muscle tissue engineering. A hyperbranched aliphatic polyester (HAP)-PTh-PCL scaffold was found to have a Young’s modulus of 59.81 kPa compared to 24.7 kPa for skeletal muscle (Jaymand et al., 2016). Other studies, however, have shown that the addition of PTh into another material caused the mechanical properties (such as the Young’s modulus) to decrease (Dias et al., 2019; Park et al., 2022). Although PTh suffers from similar issues as other CPs such as brittleness and reduction in Young’s modulus, these problems have been resolved by the addition of other biomaterials into the scaffold. Massoumi et al. found that between an electrospun chitosan-grafted-PTh scaffold and a chitosan-grafted-PTh/PCL scaffold, the scaffold with PCL supported increased biocompatibility and proliferation of HEPG2 liver carcinoma cells (Massoumi et al., 2021). Although PThs have demonstrated clear compatibility with biological tissue and skeletal muscle myoblasts and can be modified to have different functional groups, there is a dearth of research regarding myofiber alignment and large-scale tissue growth on PTh scaffolds. Additionally, the inelasticity of PTh scaffolds may pose problems for skeletal muscle growth.

## 5 Conclusion

VML is the loss of a significant volume of skeletal muscle that exceeds the natural regeneration capacity of muscle, and results in a significant and permanent loss of functionality. VML presents a significant health and financial concern in the United States. The current standard treatments include surgical transfer of muscle flaps and physical therapy. Surgical intervention is difficult and limited, while physical therapy does not induce significant muscle regeneration. Therefore, scaffolds for the regeneration of skeletal muscle are a potential solution. The ideal characteristics in scaffolds for skeletal muscle tissue engineering are: i) biocompatibility, ii) a degradation rate which aligns with the rate of tissue regeneration, iii) a three-dimensional, highly porous structure with controllable pore size, iv) alignment, and v) a Young’s modulus similar to that of skeletal muscle to withstand contractile forces, all of which contribute to the formation of myofibers.

This review highlights several methods of fabricating porous biomaterial scaffolds, such as electrospinning, porogen leaching, 3D printing, and freeze drying. Different fabrication methods form scaffolds which meet different design criteria. Control of pore size is one of the most important design criteria, as it has a notable effect on various cell types; for osteogenic cells, pores which were larger than 300 µm initiated osteogenesis of these cells, while the attachment of mesenchymal stem cells was increased upon scaffolds with 100 µm pores (Bružauskaitė et al., 2016). To advance the field of skeletal muscle regeneration, determining the ideal pore size for this application is essential to maximize myoblast infiltration and differentiation into myofibers, and to date has not been fully characterized. Alignment of a scaffold is arguably the most important criterion to meet for skeletal muscle regeneration, as skeletal muscle tissue itself is highly aligned to efficiently transmit mechanical forces. To create an ideal scaffold for VML, therefore, a fabrication method which can simultaneously be used to tune the pore size and establish alignment of the scaffold is necessary. Unfortunately, these parameters are not always explicitly analyzed, so we recommend future work to report porosity and fibrillar alignment, when possible. Electrospinning, and 3D printing are useful methods for creating alignment, while porogen leaching is useful for creating scaffolds with tunable pore sizes. Freeze drying is a method which can fulfill both of these characteristics—the pore size can be tuned, and alignment can be induced by using a constant cooling rate. 3D printing also allows for the manipulation of both pore size and alignment within scaffolds. However, care must be taken to ensure that the bulk material can retain its shape during the printing process, which can impact feature resolution within scaffolds. Based on the ability of these two methods to control both of these extremely important requirements for scaffolds for skeletal muscle regeneration, the future of scaffolds for muscle tissue engineering may lie in the development of scaffolds using either of these fabrication methods. Further research should seek to optimize both the pore size and alignment of scaffolds made using these fabrication methods.

Scaffolds made from synthetic polymers usually degrade into harmless byproducts, and conductive polymers are also generally non-toxic. It has generally been accepted that while synthetic polymers may be useful to generate scaffold architectures conducive to myofiber alignment, they lack the biological cues necessary for myoblast attachment and differentiation and therefore are almost always supplemented with natural biomaterials or peptide sequences. Although coating a synthetic scaffold with a natural material can improve bioactivity, some drawbacks may exist in the formation of this type of composite. It has been shown that coating a polypropylene mesh with ECM reduced the size of the pores in the mesh, due to accumulation of the ECM in the pores (Wolf et al., 2014). Conductive polymers are a unique and promising group to increase bioactivity of biomaterial scaffolds. Studies show that their addition to a natural or synthetic scaffold enhances the differentiation of seeded myoblasts, likely due to electrical stimulation capabilities. Creating a copolymer from a conductive and synthetic polymer has shown an increase in bioactivity as compared to the synthetic polymer alone. The same outcome has been shown using a natural material scaffold which was submerged in a conductive polymer solution. Research has shown, however, that the incorporation of conductive polymers into a scaffold may have varying effects on the porosity of the scaffold. While some studies have shown that adding a conductive polymer to a scaffold decreases overall porosity, other studies have shown that addition of a conductive polymer increases both the porosity and the surface area of the scaffold (Alegret et al., 2019). Therefore, although there are some benefits to creating composite biomaterial scaffolds, this may have detrimental effects on scaffold architecture. Further research should thus be conducted to optimize natural material coatings, potentially by decreasing the thickness of the coating. Future research should also seek to develop a method of incorporating conductive polymers into a scaffold which does not significantly affect the porosity of the scaffold.

Biomaterial sponges formed with natural biomaterials such as collagen, gelatin, alginate, chitosan, and fibrin have all shown non-toxicity, ability to support myoblast proliferation and differentiation, and advanced cell signaling capabilities. Although natural biomaterials tend to have lower stiffness than synthetic polymers, their stiffness can be easily increased using crosslinking methods. Crosslinking has been shown to increase the stiffness of collagen (Yahyouche et al., 2011), gelatin (Broderick et al., 2005), alginate (Naghieh et al., 2018), chitosan (Subramanian and Lin, 2005) and fibrin (Grasman et al., 2012) scaffolds. Therefore, the major limitation of the use of natural polymers can be overcome by the use of crosslinking. Additionally, natural biomaterial sponges with an average pore size of 100–200 µm have been shown to facilitate the formation of myotubes (Beier et al., 2009; Jana et al., 2013). We therefore suggest that further research in skeletal muscle regeneration should seek to explore crosslinked natural biomaterial scaffolds in addition to the development of natural-conductive polymer composite biomaterial scaffolds with ideal pore size for myofiber formation.

One component of tissue engineering strategies for skeletal muscle regeneration which was outside of the scope of this review is the incorporation of cells into the scaffold. There are a multitude of cell types that have been investigated for skeletal muscle tissue engineering, specifically for the treatment of VML. The reader is referred to the following review articles for discussions of cell implantation studies (Kang et al., 2020; Shayan and Huang, 2020; Eugenis et al., 2021) and cell-integrated hydrogels (McCullagh and Perlingeiro, 2015; Fischer et al., 2021). The following articles can be referenced to explore the use of cell-laden scaffolds for skeletal muscle regeneration, using cells such as satellite cells, primary myoblasts, mesenchymal stromal cells, and C2C12s (Boldrin et al., 2007; Hached et al., 2017; Gilbert-Honick et al., 2018b; Qiu et al., 2018; Nakayama et al., 2019; Gilbert-Honick et al., 2020; Narayanan et al., 2020). Future research should seek to optimize these cell-based strategies and explore their success *in vivo*.

The clinical objective for skeletal muscle tissue engineering is to induce the formation of organized muscle tissue with contraction force values similar to native muscle; this is especially important given that losses of only small parts of the muscle can result in a much larger loss of functionality. The formation of organized muscle tissue within an implanted scaffold can be induced through porosity of the scaffold, which facilitates cell infiltration, and through alignment of the scaffold, which facilitates the formation of directional myofibers. Based on current trends in research, it seems that natural biomaterial scaffolds with porosity and aligned architecture are more advantageous for muscle regeneration than scaffolds made from other biomaterial types (*i.e.,* synthetic materials). We suggest that researchers explicitly analyze and report on pore size and fibrillar alignment, where possible, to ensure accurate comparisons across scaffolds and ultimately work to enhance outcomes for skeletal muscle tissue engineering. Other promising strategies using synthetic and conductive materials have been developed, however, and show particular promise in the form of composite scaffolds that leverage the advantages of different polymer types. These strategies should continue to be explored and optimized for use in treatment of VML.
